# Inhibition of LncRNA *H19* Attenuates Testicular Torsion-Induced Apoptosis and Preserves Blood–Testis Barrier Integrity

**DOI:** 10.3390/ijms26052134

**Published:** 2025-02-27

**Authors:** Linxin Cheng, Zhibao Yin, Han Liu, Sijing Shi, Limin Lv, Yixi Wang, Meng Zhou, Meishuang Li, Tianxu Guo, Xiyun Guo, Guang Yang, Junjun Ma, Jinbo Yu, Yu Zhang, Shuguang Duo, Lihua Zhao, Rongfeng Li

**Affiliations:** 1 Jiangsu Key Laboratory of Xenotransplantation, Nanjing Medical University, Nanjing 211166, China; chenglx@stu.njmu.edu.cn (L.C.); yinzhibao@njmu.edu.cn (Z.Y.); liuhan@stu.njmu.edu.cn (H.L.); 2311210010@stu.pku.edu.cn (S.S.); mengzh10@njmu.edu.cn (M.Z.); meishuang@njmu.edu.cn (M.L.); guotianxu2021@stu.njmu.edu.cn (T.G.); guoxiyun@stu.njmu.edu.cn (X.G.); ygnjmu@stu.njmu.edu.cn (G.Y.); majunjun@stu.njmu.edu.cn (J.M.); yujinbo@stu.njmu.edu.cn (J.Y.); 2024111750@stu.njmu.edu.cn (Y.Z.); 2 State Key Laboratory of Reproductive Medicine and Offspring Health, Center for Global Health, School of Public Health, Nanjing Medical University, Nanjing 211166, China; 3 Key Laboratory of Targeted Intervention of Cardiovascular Disease, Collaborative Innovation Center for Cardiovascular Disease Translational Medicine, Nanjing Medical University, Nanjing 211166, China; gypsophilalv@163.com; 4 Laboratory Animal Center, Institute of Zoology, Chinese Academy of Sciences, Beijing 100101, China; wangyixi@ioz.ac.cn (Y.W.); duoshuguang@ioz.ac.cn (S.D.)

**Keywords:** apoptosis, blood–testis barrier, lncRNA *H19*, Sertoli cell, testicular torsion

## Abstract

Testicular torsion is a common emergency in adolescents, and can lead to severe ischemia reperfusion injury (IRI). LncRNA *H19* has been shown to increase during ischemia, but its role in testicular IRI remains unknown. Focusing on this research gap, we utilized *H19* biallelic mutant mice and Sertoli cell line (TM4) to construct in vivo and in vitro models of ischemia/reperfusion (I/R) and oxygen–glucose deprivation/reperfusion (OGD/R). Compared to WT I/R mice, *H19*^−/−^ I/R mice showed milder tissue disorganization and cell loss, with a more intact blood–testis barrier (BTB). The cell viability decreased, ROS levels and apoptosis-related factors such as Bax/Bcl-2 increased in TM4 cells after OGD/R, whereas these changes were reversed when *H19* was knocked down followed by OGD/R (si-*H19*+OGD/R). In contrast, over-expression of *H19* in TM4 cells exacerbates OGD/R-induced cell apoptosis. Through in-depth analysis of KEGG-enriched pathways, the PI3K/AKT pathway was identified as a potential target of *H19* modulation. Western blotting confirmed that, in OGD/R cells, elevated *H19* levels were accompanied by the excessive AKT phosphorylation and the tight junction marker ZO-1 degradation; and in si-*H19*+OGD/R cells, the decreased AKT phosphorylation was recovered and the up-regulated ZO-1 expression was weakened simultaneously via using the AKT activator SC79. These results suggest that inhibiting *H19* in OGD/R cells might preserve the integrity of the BTB by reversing the excessive phosphorylation of AKT. Moreover, *H19* deficiency in si-*H19*+OGD/R cells alleviated the disturbances in glycolysis, fatty acid biosynthesis, and amino acid metabolism. Our study indicates that *H19* might be a potential therapeutic target for clinic testicular I/R treatment.

## 1. Introduction

Various organs can suffer acute injury from ischemia–reperfusion (I/R), leading to conditions such as myocardial infarction, cerebral infarction, and acute kidney injury [1,2]. Testicular torsion is a common urological emergency. It happens to teenagers frequently. I/R is the main pathological foundation of testicular torsion [3]. During ischemia, the testis is affected by ischemia, hypoxia, and heat stress [4,5]. After reverse torsion treatment, ROS generation, local inflammatory response, and intracellular calcium accumulation increased [4,6]. This process is also accompanied by severe impairment of Sertoli cell function, disturbance and loss of endothelial and germ cells, sperm destruction, and testicular atrophy [7]. Most studies have demonstrated that oxidative stress plays a pivotal role in testicular torsion, such as causing germ cell arrest of cell cycle [4,8], accumulation of inflammatory factors [9], iron accumulation [10] and so on, and described various antioxidant drugs [11,12,13], anti-inflammatory drugs [14,15], and Chinese herbal medicine [16,17,18]. However, the mechanism of testicular IRI is still incomplete, especially compared with cardiovascular I/R.

Sertoli cells are one of the most complex somatic cells in the testis. Sertoli cell number and function are intimately tied to testis size and sperm production capacity [19]. As a “Nurse cell”, it provides structural and nutritional support to spermatogenic cells. They create a unique environment which allows for germ cell development and forms BTB consisting of tight junction (TJ), ectoplasmic specialization (ES), desmosomes, and gap junction (GJ) [20]. Not only does BTB provide a suitable space for spermatogenesis separating certain substances in the blood from the spermatogenic epithelium, but it also prevents sperm antigens from entering the blood to trigger an autoimmune reaction. In addition to providing structural support, Sertoli cells also exhibit endocrine and paracrine effects. For example, they mediate signal transduction between follicle-stimulating hormones (FSHs) or testosterone and germ cells. And they secrete androgen-binding protein and insulin-like growth factor I, which act directly on germ cells. These ways collectively contribute to maintaining the stability and periodicity of the spermatogenic process [19]. Moreover, the Sertoli cells also play a regulatory role in the energy metabolism of germ cells [21,22]. In the past many years, the function of Sertoli cells has been greatly underestimated. In recent years, Sertoli cells have gained a significant amount of attention, but there are few studies on the functional damage of Sertoli cells in I/R.

LncRNA *H19* is the first lncRNA to be discovered. It does not encode a protein and is highly expressed in embryos [23] and relatively low in adults; however, its expression can be increased under the circumstances of tumorigenesis, hypoxia, apoptosis, cellular inflammation and senescence, environmental stress, and so on [24]. *H19* is also an imprinted gene located in the imprinted control region 1 (ICR1) at the telomeric end of chromosome 11p15. This region is crucial for the regulation of fetal and postnatal growth and development. Hypomethylation in this region leads to reduced expression of paternal IGF2 and increased expression of maternal *H19*, which can result in growth restriction. This is one of the causes of Silver–Russell syndrome [25].

It has been shown that *H19* has a two-sided nature. For example, *H19* overexpression can improve renal function and alleviate acute kidney injury [26], as well as induce anoxia–reoxygenation injury through autophagy in hepatoma cells [27], and the inhibition of *H19* protects against cerebral IRI in rats [28]. Given these broad effects, we investigated the role of *H19* in testicular IRI. Our findings indicate that the inhibition of *H19* alleviates inflammation and apoptosis induced by testicular I/R and potentially preserves the integrity of the BTB through the PI3K/AKT/ZO-1 pathway. Consequently, we propose that targeting *H19* inhibition might be an effective therapeutic strategy for improving the prognosis of testicular IRI.

## 2. Results

### 2.1. H19 Knockout Alleviated Inflammation and Apoptosis Damage Caused by Testicular IRI

**Figure 1:**
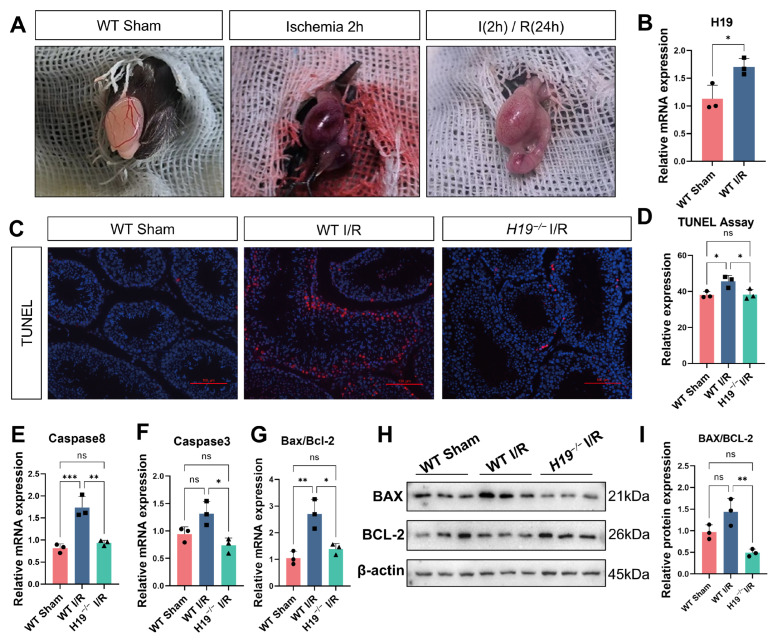
*H19* knockout alleviated apoptosis damage caused by testicular IRI. (**A**) Representative images of testicular torsion models. (**B**) Changes in *H19* mRNA expression in testis after I/R. *n* = 3 per group. (**C**) Representative images of TUNEL staining in the WT sham, WT I/R, and *H19*^−/−^ I/R mice. *n* = 3 per group, scale bars = 100 µm. (**D**) The quantification of TUNEL assay. (**E**–**G**) mRNA levels of *Caspase-8*, *Caspase-3*, and *Bax/Bcl-2* in WT Sham, WT I/R, and *H19*^−/−^ I/R. *n* = 3 per group. (**H**,**I**) The protein expressions and quantifications of Bax and Bcl-2 were assessed by Western blot in WT Sham, WT I/R, and *H19*^−/−^ I/R samples, *n* = 3 per group. Data are depicted as mean ± SD. * *p* < 0.05, ** *p* < 0.01, *** *p* < 0.001, ns, no statistical significance.

To investigate the role of *H19* in IRI, we established a testicular I/R model using adult male mice aged eight weeks or older (Figure 1A) and observed a significant increase in *H19* expression following I/R in WT mice (Figure 1B). Using TUNEL staining, we assessed apoptosis and found considerably increased apoptotic cells in WT mice after testicular torsion. However, when the same I/R model was applied to age-matched *H19*^−/−^ mice, the *H19*^−/−^ I/R mice exhibited fewer apoptotic cells than the WT I/R mice, with their apoptotic levels more closely resembling those of the WT sham mice (Figure 1C,D). The apoptosis levels in *H19*^−/−^ Sham mice were similar to those in WT sham mice (Figure S1A).

The NLRP3 inflammasome is implicated in various forms of cell death, including pyroptosis, apoptosis, necroptosis, and ferroptosis. Previous studies have linked NLRP3 activation to apoptosis in retinal ganglion cells during retinal I/R [29]. In our study, the immunofluorescence results show markedly increased expression of NLRP3 in the WT I/R mice compared to the WT sham mice, and significantly decreased levels in the *H19*^−/−^ I/R group compared to WT I/R group (Figure S1B).

Using RT-qPCR, we further examined changes in apoptotic factors at the RNA level. We observed elevated levels of *Caspase-8*, *Caspase-3*, and *Bax* long with decreased *Bcl-2* expression in WT mice after I/R. Notably, *Caspase-8* and *Bax* showed a significant increase, while *Bcl-2* exhibited a significant decrease. However, *H19*^−/−^ mice significantly reversed these changes and showed no significant changes compared to WT Sham mice (Figure 1E–G). The protein expression results detected by Western blot also showed that, after *H19* knockout, the increased Bax and decreased Bcl-2 induced by I/R were significantly improved, and apoptosis was alleviated. (Figure 1H,I).

### 2.2. H19 Knockout Diminished the Apoptotic Cell Quantities, and Improved the Sperm Motility and Organizational Structure of Testicular IRI

**Figure 2:**
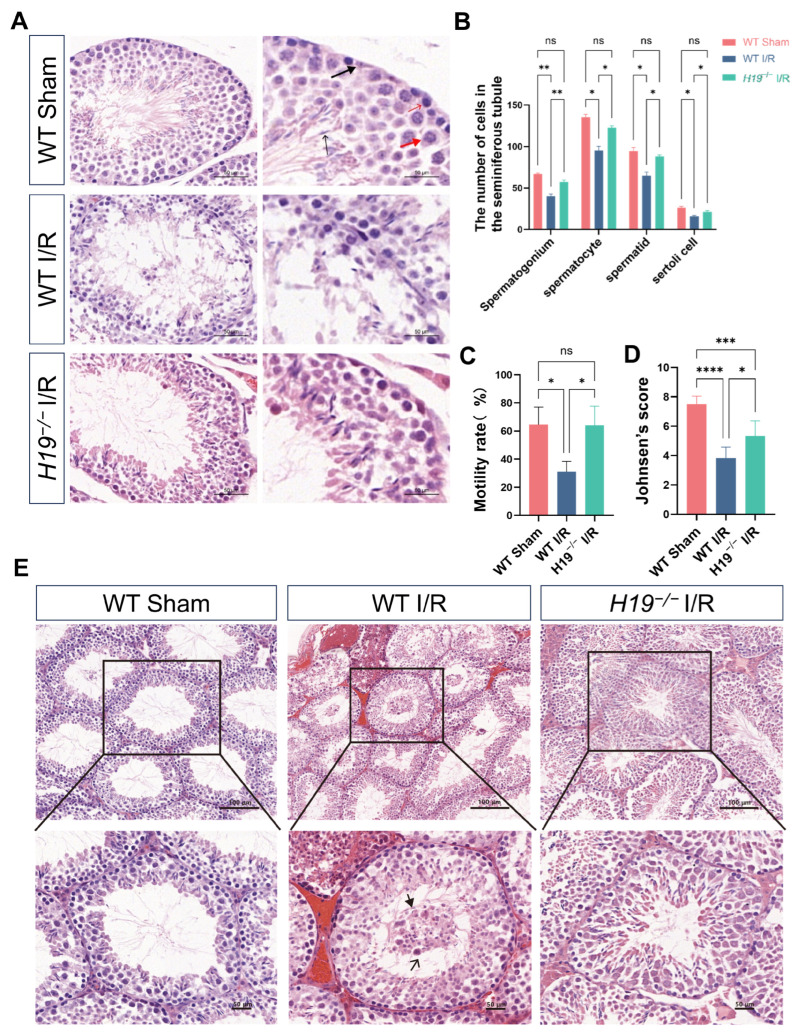
*H19* knockout diminished the apoptotic cell quantities, and improved the sperm motility and organizational structure of testicular IRI. (**A**,**B**) Changes in the number of various cell types in the testes. The thin black arrow indicates spermatid, the thin red arrow indicates spermatogonium, the black thick arrow indicates purple Sertoli cells, and the red thick arrow indicates spermatocyte undergoing meiosis. *n* = 3 per group. Scale bars = 50 µm. *n* = 3 per group. (**C**) Comparison of sperm motility analysis. *n* = 2–5 per group. (**D**) Johnsen’s score was evaluated 2 h after testicular torsion and 24 h reperfusion. *n* = 6 per group. (**E**) HE staining and analyses of testes obtained from WT sham, WT I/R, and *H19*^−/−^ I/R groups. The thick arrow indicates a decrease in mature sperm, the thin arrow indicates the shedding of spermatogenic cells into the lumen. *n* = 3 per group. Scale bars = 50 µm, 100 µm. Data are depicted as mean ± SD. * *p* < 0.05, ** *p* < 0.01, *** *p* < 0.001, **** *p* < 0.0001, ns, no statistical significance.

To assess the impact of testicular torsion on various cell types within the testis, we performed HE staining on the WT sham, WT I/R, *H19*^−/−^ sham, and *H19*^−/−^ I/R groups of mice. The HE staining of *H19*^−/−^ sham and WT sham group mice revealed similar testicular structures (Figure S1C). Compared to WT sham mice, WT I/R mice exhibited markedly decreased cell numbers, such as spermatogonia, spermatocytes, spermatids, and Sertoli cells, and disorganized arrangements within the seminiferous tubules (Figure 2A,B). Sperm motility and Johnsen’s score were also considerably reduced (Figure 2C,D). Pathomorphological analysis revealed seminiferous tubules with irregular boundaries, disarranged cells, shedding of seminiferous cells to varying degrees, aggravated edema, and minor infiltration of inflammatory cells in WT I/R mice compared to WT sham mice. Degenerated germ cells were observed to shed into the lumen (thin arrow), and the mature sperm count decreased (thick arrow) (Figure 2E). In contrast, *H19*^−/−^ I/R mice exhibited cellular distributions resembling WT sham mice, with relatively normalized sperm motility and Johnsen’s score, and more orderly spermatogenic cell arrangements, except for a few exfoliated cells seen in the lumen in Figure 2C–E.

### 2.3. H19 Knockout Maintained the Integrity of the BTB

The blood–testis barrier (BTB) is crucial for spermatogenesis in males. It comprises TJs, adherens junctions between Sertoli cells, specialized extracellular matrix components, the basement membrane, and other elements. These components create an essential microenvironment for the development of germ cells. ZO-1, Occludin, and various Claudins are key components of intercellular TJs. N-Cadherin and E-Cadherin are the main components of the ES, while α-SMA is a major constituent of the testicular stromal membrane. The degradation of these components can lead to the disruption of BTB integrity, increased permeability, and subsequent testicular damage [30].

**Figure 3:**
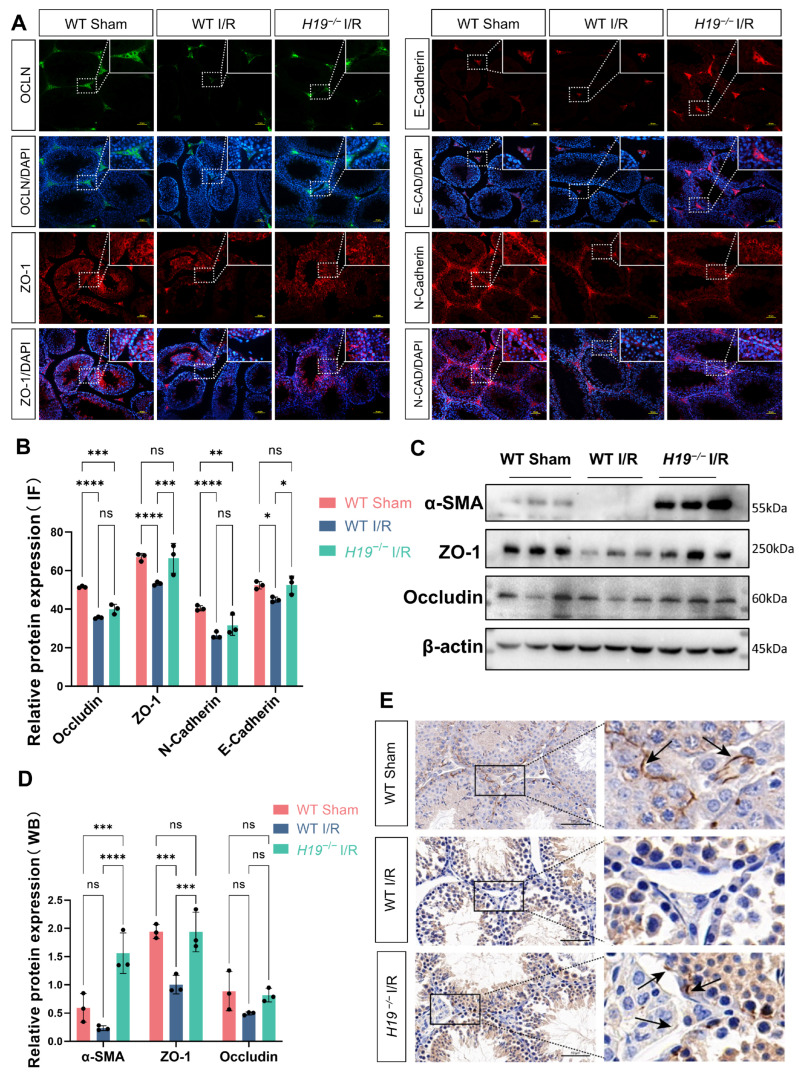
*H19* knockout maintained the integrity of the BTB. (**A**,**B**) Representative images and quantifications of Occludin, ZO-1, E-Cadherin and N-Cadherin expression in WT Sham, WT I/R and *H19*^−/−^ I/R analyzed by immunofluorescence (Occludin, ZO-1, E-Cadherin, and N-Cadherin were located in cell membrane and cell junctions. Green indicates mouse primary antibodies, and red indicates rabbit primary antibodies). *n* = 3 per group. Scale bars = 50 µm. (**C**,**D**) The protein expressions and quantifications of α-SMA, ZO-1 and Occludin were assessed by Western blot in WT sham, WT I/R, and *H19*^−/−^ I/R mice, *n* = 3 per group. (**E**) Immunohistochemical expression of ZO-1 in WT sham, WT I/R, and *H19*^−/−^ I/R mice (black arrows indicate the locations of ZO-1 expression). Scale bars = 50 µm. Data are depicted as mean ± SD. * *p* < 0.05, ** *p* < 0.01, *** *p* < 0.001, **** *p* < 0.0001, ns, no statistical significance.

To assess barrier integrity, we examined these proteins related to BTB via immunofluorescence and found that Occludin (OCLN), ZO-1, N-Cadherin, and E-Cadherin levels markedly decreased in the WT I/R group compared to the WT sham group (Figure 3A,B). Western blot also showed the substantially declined α-SMA, ZO-1 and Occludin (Figure 3C,D). These all indicate a rise in BTB permeability and a weakening of integrity, which are undesirable outcomes. Notably, *H19*^−/−^ I/R mice exhibited a more intact BTB compared to the WT I/R group and their testicular-related protein expression is more similar to that of the WT sham group. By immunohistochemistry, we detected the expression of ZO-1. In WT mice, ZO-1 was primarily localized at the junctions of Sertoli cells near the basement membrane. In WT I/R mice, ZO-1 exhibited a scattered distribution and even disappeared from its original location. However, in *H19*^−/−^ I/R mice, ZO-1 restored its original expression pattern (Figure 3E). In summary, *H19* knockout significantly alleviated BTB damage caused by I/R.

### 2.4. Treatment with OGD/R-Induced Inflammation Response, Apoptosis, and Peroxidation in TM4 Cells

**Figure 4:**
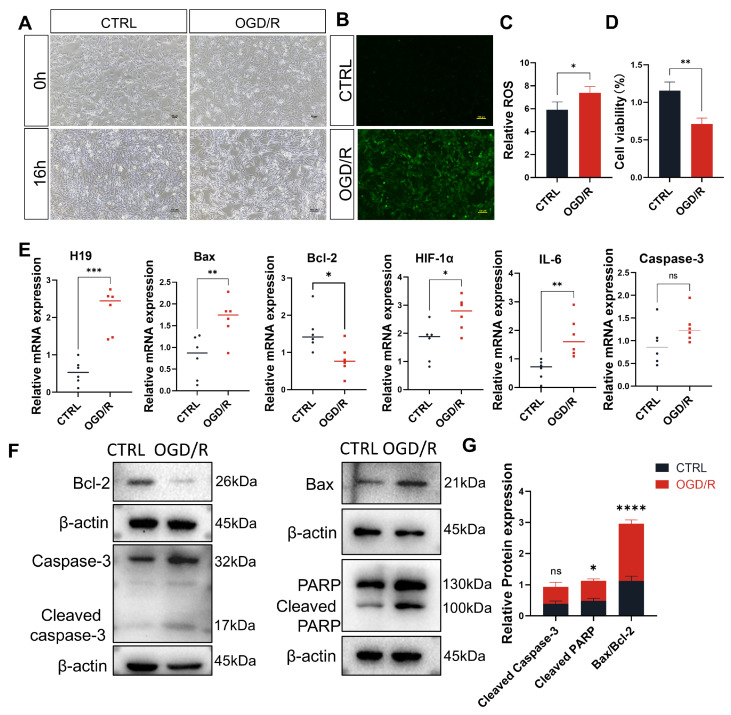
Treatment with OGD/R induced inflammation response, apoptosis, and peroxidation in TM4 cells. (**A**) Representative images of the CTRL and OGD/R groups at 0 h and 16 h of treatment; Scale bars = 100 µm. (**B**) ROS green fluorescence in the CTRL and OGD/R groups at 16 h treatment under fluorescence microscopy. *n* = 3 per group. Scale bars = 100 µm. (**C**) Relative ROS expression levels were detected by a microplate reader. *n* = 3 per group. (**D**) CCK-8 viability assay for the CTRL and OGD/R groups. *n* = 3 per group. (**E**) qPCR analysis and comparison of *H19*, *Bax*, *Bcl-2*, *HIF-1α*, *IL-6*, and *Caspase-3* in CTRL and OGD/R groups through six biological replicates. (**F**,**G**) Cleaved Caspase-3, Cleaved PARP, Bax, and Bcl-2 protein levels and quantitation in TM4 cells before and after OGD/R. *n* = 3 per group. Data are depicted as mean ± SD. * *p* < 0.05, ** *p* < 0.01, *** *p* < 0.001, **** *p* < 0.0001, ns, no statistical significance.

To validate our findings in animal experiments and delve into the underlying mechanisms, we conducted in vitro experiments using TM4 cells. We established an OGD/R model for TM4 cells using a Mitsubishi anaerobic bag and low-glucose medium. Following 4 h of hypoxia, the cells exhibited elongation and increased cell death. Even after subsequent recovery with oxygen and glucose, the cells maintained an elongated, branching morphology, with reduced growth rates compared to the CTRL group (Figure 4A).

To evaluate cellular oxidative stress levels, we performed ROS assays, which revealed increased ROS generation in OGD/R TM4 cells compared to the CTRL TM4 cells (Figure 4B,C). We also assessed cell proliferation and observed a significant reduction in proliferation capacity following OGD/R compared to the CTRL group (Figure 4D).

The results of the quantitative RT-PCR indicate that the levels of lncRNA *H19* in TM4 cells significantly increased after OGD/R, with a marked increase in the pro-apoptotic factor *Bax* and a significant decrease in the anti-apoptotic factor *Bcl-2*. These findings are consistent with the results observed in WT mice subjected to I/R in the animal experiments. Additionally, the hypoxia-inducible factor *HIF-1α* and the inflammatory cytokine *IL-6* were also greatly upregulated, while *Caspase-3* showed no significant change at the RNA level (Figure 4E). Western blot analysis of protein levels revealed that Bax, Cleaved Caspase-3, and Cleaved PARP were up-regulated in TM4 cells after OGD/R, with Bax and Cleaved PARP showing particularly significant increases, while Bcl-2 was significantly down-regulated (Figure 4F,G). These results collectively highlight the dysregulation of apoptotic and inflammatory pathways, along with increased oxidative stress in OGD/R TM4 cells.

### 2.5. The Inhibition of *H19* Contributed to Easing Inflammation, Apoptosis, and Oxidative Stress in OGD/R TM4 Cells

To further investigate the role of *H19* in testicular I/R, we designed siRNA targeting *H19*. Transfection of TM4 cells with si-*H19* resulted in less severe damage (Figure S2A). Detailed sequences and the effects of si-*H19* are presented in Supplementary Figures (Figure S2B,C). The transfection with si-*H19* was conducted 24 h prior to inducing hypoxia, followed by 4 h of hypoxia and 12 h of reperfusion to establish the OGD/R model (Figure S2D).

**Figure 5:**
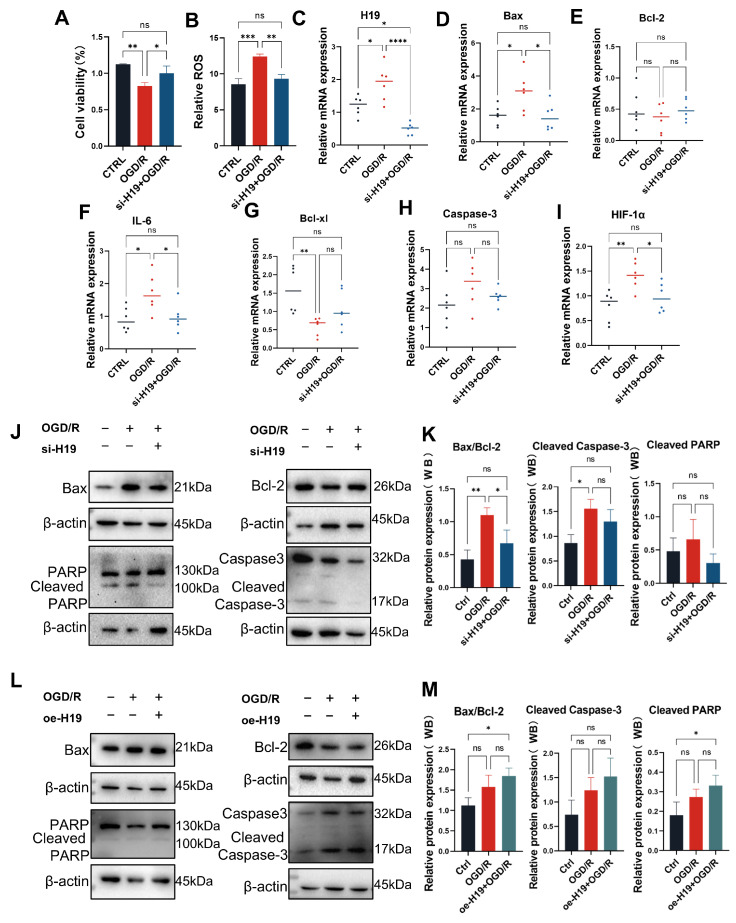
The inhibition of *H19* contributed to easing inflammation, apoptosis, and oxidative stress in OGD/R TM4 cells. (**A**) Cell viability of CTRL, OGD/R, and si-*H19*+OGD/R TM4 cells. *n* = 3 per group. (**B**) ROS generations of CTRL, OGD/R, and si-*H19*+OGD/R TM4 cells. *n* = 3 per group. (**C**–**I**) Expression of *H19*, *HIF-1α*, *Caspase-3*, *Bax*, *Bcl-2*, *IL-6*, and *Bcl-xl* mRNA by RT-qPCR in CTRL, OGD/R, and si-*H19*+OGD/R through six biological replicates. (**J**,**K**) Protein expressions and quantifications of Cleaved Caspase-3, Cleaved PARP, Bax, and Bcl-2 by Western blot in CTRL, OGD/R, si-NC+OGD/R, and si-*H19*+OGD/R TM4 cells. *n* = 3 per group. (**L**,**M**) Protein expressions and quantifications of Cleaved Caspase-3, Cleaved PARP, Bax, and Bcl-2 by Western blot in CTRL, OGD/R, oe-NC+OGD/R and oe-*H19*+OGD/R TM4 cells. *n* = 3 per group. Data are depicted as mean ± SD. * *p* < 0.05, ** *p* < 0.01, *** *p* < 0.001, **** *p* < 0.0001, ns, no statistical significance.

For CTRL, OGD/R and si-*H19*+OGD/R groups, we performed CCK-8 assay and the result indicated higher cell viability in the si-*H19*+OGD/R group than the OGD/R group (Figure 5A). ROS detection showed that ROS generation in the si-*H19*+OGD/R group was less pronounced compared to the OGD/R group (Figure 5B). Furthermore, qPCR analysis revealed that the up-regulation of *H19*, *HIF-1α*, *Bax*, and *IL-6* was substantially alleviated following *H19* knockdown and OGD/R compared to OGD/R group, whereas the down-regulation of *Bcl-2* and *Bcl-xl* was also greatly mitigated. The RNA level of *Caspase-3* showed no significant change (Figure 5C–I). Western blot analysis further demonstrated that the inhibition of *H19* alleviated the abnormal up-regulation of pro-apoptotic factors Bax, Cleaved Caspase-3, and Cleaved PARP, as well as the abnormal down-regulation of the anti-apoptotic factor Bcl-2 (Figure 5J,K).

To further confirm the role of *H19* in testicular IRI, we synthesized the gene for lncRNA *H19* and ligated its entire sequence into a pcDNA3.1 vector before transfecting TM4 cells. Quantitative PCR confirmed significant up-regulation of *H19* expression (Figure S2E) Subsequently, we induced the OGD/R model in both the CTRL and pcDNA3.1-*H19* (oe-*H19*) groups and assessed protein levels of Bax, Bcl-2, Cleaved PARP, and Cleaved Caspase-3. The results indicate higher levels of Bax/Bcl-2, Cleaved PARP, and Cleaved Caspase-3 in the oe-*H19*+OGD/R group compared to the OGD/R group (Figure 5L,M).

### 2.6. The RNA Sequencing Showed That the Elevation of *H19* Was Accompanied by Excessive Phosphorylation of AKT

**Figure 6:**
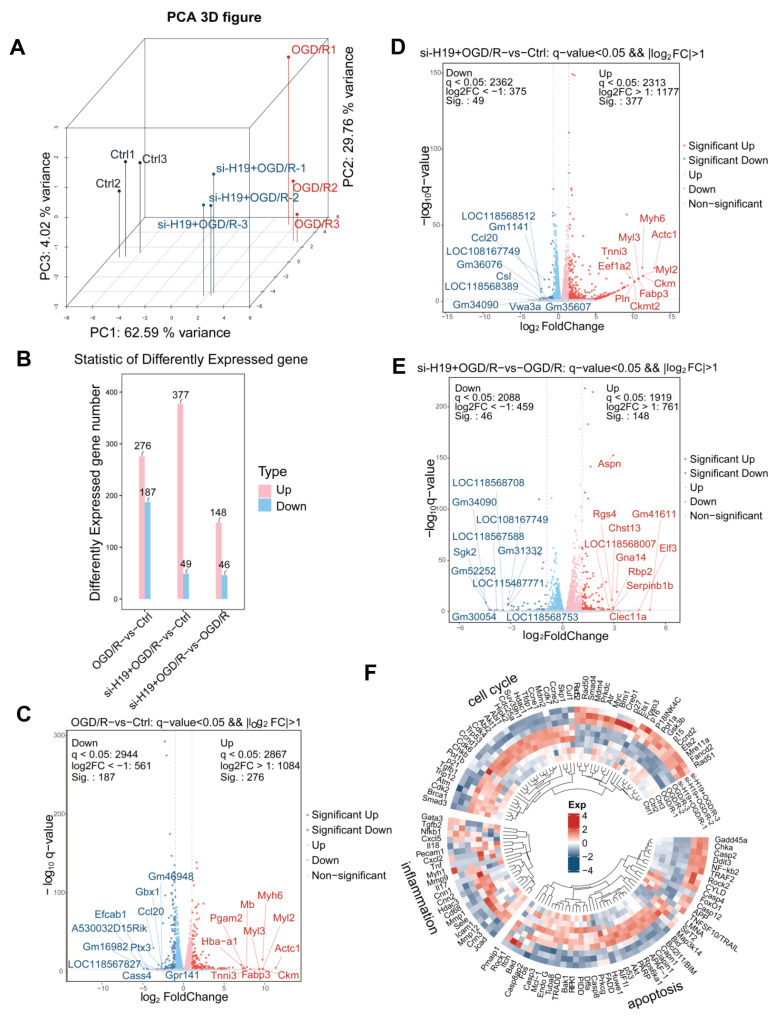
RNA-Seq analysis of CTRL, OGD/R, and si-*H19*+OGD/R groups. (**A**) Global gene expression profiling of CTRL, OGD/R, and si-*H19*+OGD/R. (**B**–**E**) Statistic and comparison of different expressed genes of CTRL, OGD/R, and si-*H19*+OGD/R. (**F**) Expression heatmap of apoptosis, inflammation, and cell cycle markers in CTRL, OGD/R, and si-*H19*+OGD/R. *n* = 3 per group.

To validate the results from both animal and cell experiments and discern molecular differences among distinct experimental conditions, we conducted transcriptome sequencing on CTRL, OGD/R, and si-*H19*+OGD/R groups. The PCA results of the transcriptome analyses showed that three groups were separated by the PC1 and PC2 dimensions (Figure 6A). In comparisons of OGD/R-vs-CTRL, si-*H19*+OGD/R-vs-CTRL, and si-*H19*+OGD/R-vs-OGD/R, we observed 276, 377, and 148 up-regulated genes, and 187, 49, and 46 down-regulated genes, respectively (Figure 6B). We extracted the top 10 genes that showed increased and decreased expression in each pairwise comparison, and a significant number of these gene sets associated with cardiomyopathy (Figure 6C–E). Furthermore, we generated cluster heatmaps for apoptosis-related factors, inflammation-related factors, and cell cycle-related factors, revealing reduced apoptosis and inflammatory responses in the si-*H19*+OGD/R group compared to the OGD/R group. While cell cycle dynamics were affected in both groups, the impact was notably lesser in the si-*H19*+OGD/R group than the OGD/R group (Figure 6F). Enrichment analysis using Wiki pathway highlighted a significant elevation in oxidative stress response following OGD/R, whereas *H19* knockdown substantially attenuated both oxidative stress and oxidative damage responses (Figure S3A,B).

**Figure 7:**
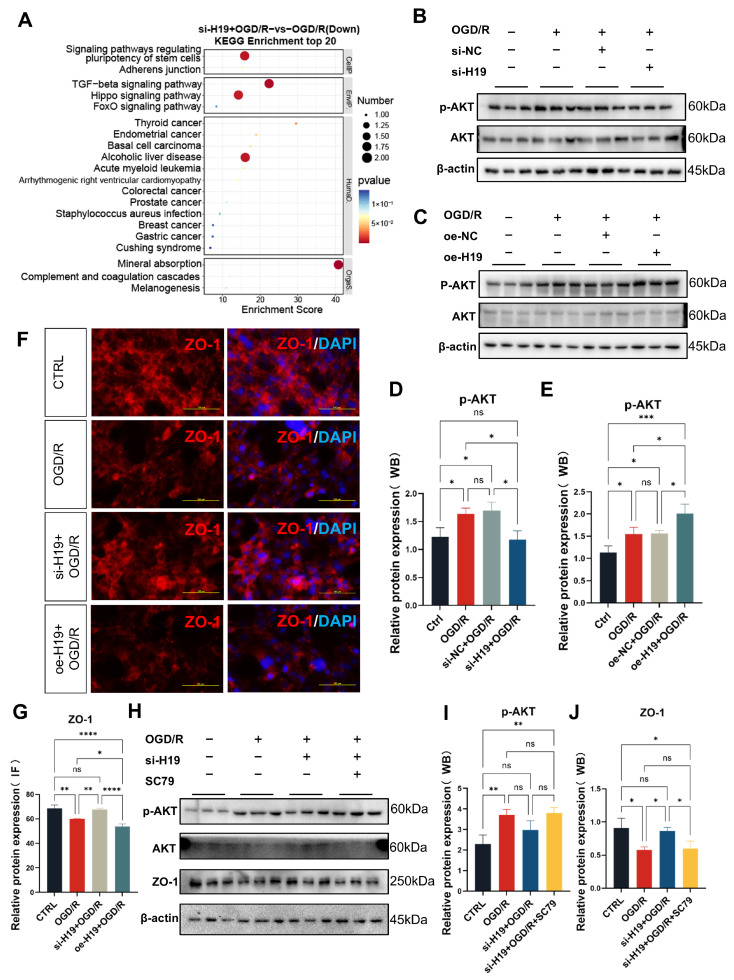
Inhibition of *H19* preserves ZO-1 expression possibly by preventing AKT excessive phosphorylation in OGD/R TM4 cells. (**A**) KEGG enrichment and comparison between the si-*H19*+OGD/R and the OGD/R TM4 cells. (**B**) Protein expression of p-AKT and AKT in the CTRL, OGD/R, si-NC+OGD/R, and si-*H19*+OGD/R TM4 cells. (**C**) Protein expression of p-AKT and AKT in the CTRL, OGD/R, oe-NC+OGD/R, and oe-*H19*+OGD/R TM4 cells. (**D**,**E**) The quantifications of (**B**,**C**). (**F**,**G**) Representative images and quantifications of ZO-1 in TM4 cells of CTRL, OGD/R, si-*H19*+OGD/R, and oe-*H19*+OGD/R analyzed by immunofluorescence (ZO-1 is localized at the cell membrane and cell junctions). *n* = 3 per group. Scale bars = 100 µm. (**H**–**J**) Western blot analysis and quantifications were performed to analyze the changes in p-AKT, AKT, and ZO-1 in CTRL, OGD/R, si-NC+OGD/R, and si-*H19*+OGD/R. *n* = 3 per group. Data are depicted as mean ± SD. * *p* < 0.05, ** *p* < 0.01, **** *p* < 0.0001, ns, no statistical significance.

Interestingly, in the si-*H19*+OGD/R group, significant reductions were observed in pathways related to stem cell development, TGF-β signaling, and Hippo signaling compared to the OGD/R group, indicating potential modulation by *H19* (Figure 7A). Further analysis using KEGG indicated a convergence towards the AKT signaling pathway across these enriched pathways, suggesting a pivotal role of AKT in mediating the observed effects. Through Western blot, we validated the AKT pathway and found up-regulation of phosphorylated AKT in the OGD/R group compared to CTRL group, down-regulation in the si-*H19*+OGD/R group, and up-regulation more in the oe-*H19*+OGD/R group (Figure 7B–E), which demonstrated the potential mechanism by which *H19* works.

### 2.7. Inhibition of *H19* Preserves ZO-1 Expression Possibly by Preventing AKT Excessive Phosphorylation in OGD/R TM4 Cells

Through Wiki pathway and Gene Set Enrichment Analysis (GSEA), we observed an up-regulation of the Matrix Metalloproteinases (MMPs) pathway in the OGD/R group to CTRL group, potentially linked to BTB impairment. Interestingly, this pathway’s elevation was attenuated after *H19* knockdown and OGD/R (Figure S3C–E). To assess the integrity of the BTB, we examined ZO-1 expression via cell fluorescence in various experimental groups: CTRL, OGD/R, si-*H19*+OGD/R, and oe-*H19*+OGD/R. The results indicate a significant decrease in ZO-1 in the OGD/R group compared to the CTRL group, indicative of BTB disruption in TM4 cell OGD/R. Notably, *H19* knockdown substantially reversed this decrease, and over-expression of *H19* exacerbated ZO-1 reduction (Figure 7F,G).

Furthermore, we explored whether AKT activation contributed to the decrease in ZO-1 expression. Using the AKT activator SC79, we observed that its application in the si-*H19*+OGD/R group led to further reduced ZO-1 expression (Figure 7H–J). These findings suggest a potential role of AKT signaling in mediating the effects of *H19* on the BTB integrity under OGD/R conditions.

### 2.8. Inhibition of *H19* May Help Maintain Energy Metabolism in TM4 Cells

In addition, we observed changes in glucose metabolism in TM4 cells. Sertoli cells absorb glucose through glucose transporters (GLUT), in particular GLUT1 and GLUT3, and convert it to lactate through LDH. LDHA catalyzes the conversion of pyruvate and NADH to lactic acid and NAD, while LDHB protein facilitates the reversible conversion of between lactate and pyruvate [31]. It is then exported via a specific monocarboxylate transporter (MCT). This exogenous lactic acid is the energy that the developing germ cell needs, and the lactate supply regulates the survival and metabolic activity of the spermatocyte [32]. The cells themselves rely on fatty acid oxidation for energy maintenance [33]. Fatty acid β oxidation is considered as the major energy source of Sertoli cell. Some amino acids also provide energy to SC in the body by participating in the TCA cycle [22]. Therefore, the energy metabolism of Sertoli cells is necessary for spermatogenesis.

**Figure 8:**
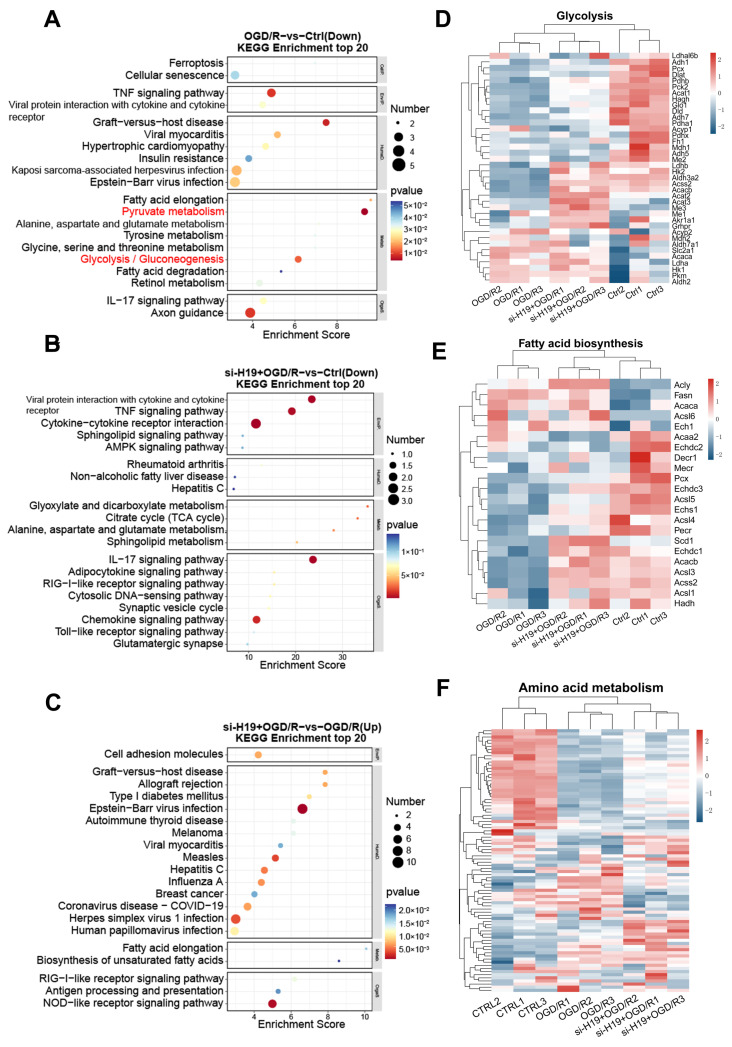
Inhibition of *H19* may help maintain energy metabolism in TM4 cells. (**A**–**C**) KEGG enrichment and comparative analysis of CTRL, OGD/R, and si-*H19*+OGD/R. (**D**–**F**) Heatmap of gene expression related to glycolysis, fatty acid biosynthesis, and amino acid metabolism. *n* = 3 per group.

However, the effect of I/R on energy metabolism in Sertoli cells is unclear, as is the effect of *H19* knockout in their metabolic capacity. KEGG analysis of the transcriptome revealed a decline in several energy metabolism-related pathways in the OGD/R and si-*H19*+OGD/R groups compared to the CTRL group, including fatty acid elongation, pyruvate metabolism, and glycolysis, etc. Notably, pyruvate metabolism and glycolytic pathways were most enriched in the OGD/R group compared to the CTRL group (Figure 8A–C). Disruption of pyruvate metabolism impairs glucose metabolism and lactate production, which is critical for germ cell function [31].

By enriching glycolysis-related genes, we found that ldhal6b, hk2, ldhb, and so on were down-regulated in OGD/R TM4 cells, while these genes partially returned to normal levels following *H19* inhibition. A similar conclusion was drawn from the analysis of fatty acid biosynthesis-related genes, suggesting that *H19* inhibition stabilizes the metabolic disorders induced by OGD/R. Although the effects on amino acid metabolism were less pronounced, most genes with abnormal up-regulation or down-regulation showed slight mitigation after *H19* inhibition (Figure 8D–F).

## 3. Materials and Methods

### 3.1. Animals

All mice were housed in barrier facility with a 12 h light/dark cycle. All animal experiments were approved by the Institutional Animal Care and Use Committee of the Institute of Zoology, Chinese Academy of Sciences (CAS). All efforts were made to minimize animal suffering and reduce the number of animals used.

### 3.2. Generation of *H19*^−/−^ Mice

The *H19*^+/−^ mice were generated through CRISPR/Cas9-mediated genome editing. The procedure involved the in vivo recovery of embryo followed by pronuclear microinjection of the CRISPR/Cas9 components. The resulted *H19*^+/−^ mice were then bred to produce both male and female *H19*^−/−^ mice. The position and sequence of the sgRNA, as well as the genotypes of the wild type and *H19* knockout, are shown in Figure S4A. The results of the mouse genotyping identified by RT-PCR and agarose gel electrophoresis are shown in Figure S4B. Some male *H19*^−/−^ mice were directly used in experiments and the other mice were used for sperm collection and cryopreservation for in vitro fertilization with *H19*^+/−^ or *H19*^−/−^ female mice-derived oocytes followed by embryo transfer to produce more male *H19*^−/−^ mice.

### 3.3. Establishment of the Testis I/R Model

The mice were anesthetized by intraperitoneal injection of Avertin (1.25% *w*/*v* tribromoethanol) at a dose of 0.2 mL/10 g body weight. Along the midline of the abdomen, a vertical incision was made, the testicular fat was located, and the left testis was pulled [9]. Slightly separate the fat and connective tissue around the testis and exposing spermatic cord and its blood vessels, then rotate 720° clockwise and fix in place with a vascular clamp to induce ischemia. We can notice the testis gradually reaching a congestion-related purple color. After 2 h, the microvascular clamp was released and the blood flow of the testis was gradually restored. After 24 h, the mice were euthanized, and the testes were taken out or put into liquid nitrogen or fixed liquid for further tests.

### 3.4. HE Staining and Cell Counting

Fresh testes at the same developmental stage were isolated and fixed in MDF fixative solution (30% formaldehyde, 15% ethanol, 5% glacial acetic acid, and 50% distilled water), replaced with 10% paraformaldehyde after 24 h, and then treated with dehydration and paraffin embedding. The tissues were sectioned into paraffin sections about 5 μm thick, dewaxed, rehydrated, and stained with hematoxylin–eosin (HE). Then, they were analyzed under an light microscope. In the process of the analysis, different cell types were identified. High-resolution images of the sections were captured, and the spermatogonia, spermatocytes, splightermatids, and Sertoli cells in the seminiferous tubules were counted.

### 3.5. Johnsen’s Score

Under a light microscope, at least 50 cross-sections of seminiferous tubules were randomly observed, and each tubule was scored according to the Johnsen scoring criteria. A 10-point score indicates normal seminiferous tubule structure with distinct layers of all-level spermatogenic cells, normal sperm production, and numerous mature sperm in the lumen. A 9-point score means the spermatogenic cell layers are basically intact with a slight reduction in sperm production but still many mature sperm. An 8-point score shows relatively intact spermatogenic cell layers with a further decrease in sperm production and a significant drop in mature sperm numbers. At 7 points, the spermatogenic cell layers are reduced, spermatids decrease, and sperm production notably declines. When it is 6 points, the spermatogenic cell layers are fewer, mainly spermatocytes, with few spermatids and almost no mature sperm. A 5-point score implies a significant reduction in spermatogenic cell layers, only a small number of spermatocytes, and rare spermatids. At 4 points, there is a marked reduction in spermatogenic cells, only Sertoli cells, and a few spermatogonia, lacking spermatocytes and spermatids. A 3-point score represents the Sertoli cell-only syndrome where only Sertoli cells are in the seminiferous tubules. At 2 points, both spermatogenic and Sertoli cells in the seminiferous tubules are reduced, and the lumen is atrophied. Finally, a 1-point score indicates severely atrophied seminiferous tubules with thickened walls and no spermatogenic or Sertoli cells.

### 3.6. Immunofluorescence Staining

Fresh tissue was removed and fixed successively in MDF and 10% paraformaldehyde, at 4 °C overnight and then embedded. The embedded wax blocks were cut into 5 μm sections and dried in a 60 °C oven. After the xylene, anhydrous ethanol, 90% ethanol, 85% ethanol, 80% ethanol, 75% ethanol, and water were added to achieve the sequential soaking dewaxing process, antigen retrieval was performed by microwaving them on low for 20 min. We completed the 30 min ice bath cooling, 20 min transparentizing and 1 h antigen blocking before treating them with the primary antibody at 4 °C overnight. The second day, the sections were washed three times with PBS, and treated with secondary antibody at room temperature for one hour. The primary antibodies included the following:NLRP3, OCCLUDIN, ZO-1, N-Cadherin, E-Cadherin; The secondary antibodies included Cy3-conjugated goat anti-rabbit IgG (H+L), goat anti-mouse IgG Alexa Fluor 488 (The detailed information of the antibodies is provided in Supplementary Table S2). We washed them three times with PBS before staining with DAPI. We observed fluorescence intensity under the microscope.

### 3.7. Immunohistochemical Staining

After fixing and embedding the tissue, 5 μm sections were prepared. The paraffin sections were deparaffinized in xylene and rehydrated through a series of graded ethanol to water. To quench endogenous peroxidase activity, 3% hydrogen peroxide was applied, followed by high-temperature and high-pressure antigen retrieval using citrate buffer. The sections were blocked with goat serum to reduce nonspecific binding, and the primary antibody was added and incubated overnight at 4 °C. The next day, the sections were washed and incubated with the secondary antibody. DAB was used for color development. Finally, the nuclei were counterstained with hematoxylin, followed by rinsing, dehydration, clearing, and mounting.

### 3.8. Sperm Counting and Motility Assay

The epididymis and testes were dissected from the mice, and three incisions were cut into the tail of epididymis, to which we then added 200 μL of pre-equilibrated (37 °C) droplet of DMEM medium (Gibco, Suzhou, China; C11995500BT) supplemented with 10% FBS. Sperm swam out from the tail of the epididymis and capacitated in 37 °C for 5 min. Then, 10 μL of sperm sample was collected and placed on a counting plate. We used the CASA instrument to observe and take photos and issue reports.

### 3.9. TUNEL Assay

Apoptosis in mouse testis was evaluated with the TUNEL cell apoptosis detection kit (Beyotime, Shanghai, China; C1089). According to the instructions of the reagent, 20 μg/mL proteinase K without DNase was added to the paraffin sections after dewaxing for 30 min at room temperature. Sections were washed three times, to which were added 50 μL of TUNEL solution, and incubated in 37 ° C incubator for 60 min. Finally, we observed the fluorescence under the fluorescence microscope.

### 3.10. Cell Culture and Viability Assay

The TM4 cell line was purchased from Cell Bank of the Chinese Academy of Sciences and cultured in Dulbecco’s Modified Eagle’s medium (DMEM/F12) supplemented with 2.5% fetal bovine serum, 5% horse serum, and 1% penicillin/streptomycin at 37 °C under a humified atmosphere consisting of 95% air and 5% CO_2_. The CCK-8 kit (ABCLONAL, Wuhan, China; RM02823) was used to assess the viability of the TM4 cells. The cells were seeded in 96-well plates at a density of 1 × 10^4^ cells per well in 100 μL of medium and then cultured for 24 h; the cells were then transfected and treated with OGD/R. The control group was cultured in normal medium for 64 h. After this, 10 μL CCK-8 solution was added to each well and incubated continuously for 2 h at 37 °C. The absorbance was measured at a 450 nm wavelength using a microplate reader (BIOTCK, Gene Company limited, Hong Kong, China). Three independent experiments were performed.

### 3.11. Establishment of the OGD/R Model

The OGD/R model was established by replacing the culture medium with glucose-low DMEM (Gibco, USA; 11054-020) after washing cells twice with PBS (Gibco). Cells were then placed in an anaerobic box with Mitsubishi anaerobic bag (JAPAN) containing less than 1% oxygen at 37 °C for 4 h. Control cells were incubated in serum-free medium supplemented with 4.5 g/L D-glucose under normal conditions (5% (*vol*/*vol*) CO_2_ and 95% (*vol*/*vol*) air) for the same duration. At the end of the exposure period, the cells were returned to normal conditions and incubated with serum-free medium supplemented with glucose for 12 h. Six independent experiments were performed.

### 3.12. siRNA and Transfection

*H19* siRNA was synthesized by RIBOBIO, and the transfection reagent (EKBIO, Nanjing, China; EK-23002) was used to transfect the *H19* siRNA. After the cells were cultured for 24 h, the transfection reagent and siRNA were diluted with basal medium according to the instructions of the transfection reagent, so that the final concentration was 60 nm, at least 24 h after transfection to ensure the efficiency of cell transfection.

### 3.13. Transfection of *H19* Overexpression Plasmid

The lncRNA *H19* overexpression vector was synthesized by Shanghai Genscript and inserted into the pcDNA3.1-3 × Flag vector available in our laboratory. The full length of the gene was 2288 bp.

### 3.14. Activation of AKT Pathway

SC79 powder (TargetMol, USA; T2274) was dissolved in DMSO to a concentration of 25 mg/mL. Two hours before the end of OGD/R, SC79 was added to the target culture dish to a final concentration of 12 μg/mL to activate the AKT pathway.

### 3.15. Quantitative Real-Time PCR Analysis

Total RNA was extracted from the testis and cultured TM4 cells using the Total RNA Kit (Promega, Beijing, China; LS1040) according to the manufacturer’s instructions. The cDNA was then synthesized using HiScriptII Q RT Super Mix (Vazyme, Nanjing, China) and was used for Quantitative Real-Time PCR (qPCR) reactions. qPCR was performed using ChamQ SYBR qPCR Master Mix (Vazyme, Nanjing, China; Q341) on a 96-well optical reaction plate in 7500 Real-time System (Applied Biosystems, Waltham, MA, USA). β-actin was used as an internal control to standardize $2^{-\Delta \Delta \mathrm{CT}}$ values of target genes. The primer sequences are shown in Table S1. Each biological repeat was in sextuplicate and on different days.

### 3.16. Western Blotting

Total proteins were extracted from the testis or cultured TM4 cells using RIPA lysis buffer supplemented with protease inhibitors. Protein concentrations were measured using PierceTM BCA protein assay (Thermo Fisher Scientific, Waltham, MA, USA; 23227). Protein samples were separated on 10% or 7.5% (*wt*/*vol*) SDS-polyacrylamide gels and then transferred to PVDF membranes. The membranes were blocked with 5% nonfat milk for 2 h and incubated with primary antibody overnight at 4 °C. The next day, the membranes were washed three times with TBST, then incubated with secondary antibody for 2 h. The primary antibodies included the following: Bax, Bcl-2, Cleaved Caspase-3, Cleaved PARP, Occludin, ZO-1, α-SMA, β-actin, p-AKT, AKT. The secondary antibodies included goat anti-rabbit IgG, goat anti-mouse IgG (The detailed information of the antibodies is provided in Supplementary Table S2). The expression of target proteins was normalized to β-actin obtained from the same sample (taken as 1.0) and then quantified using ImageJ Software (version 1.54g, Wayne Rasband and contributors, National Institutes of Health, Bethesda, MD, USA). At least three independent experiments were performed.

### 3.17. ROS Levels

The ROS levels were examined using Reactive Oxygen Species Assay Kit of Beyotime. After treatment, DCFH-DA was added the cells of different groups at the same time, and Rosup as positive control was added in the normal cells for 30 min at 37 °C. The mean fluorescence intensity was detected with a fluorescence microscope. For absorbance examinations, the TM4 cells were harvested and detected with a Microplate Reader.

### 3.18. RNA-Seq

The RNA libraries for TM4 cells were sequenced by OE Biotech, Inc., Shanghai, China. Bioinformatic analysis was performed using the OECloud tools at https://cloud.oebiotech.com/task/, accessed on 5 March 2024. The cluster heatmap was drawn based on the R (https://www.r-project.org/, accessed on 5 March 2024) on the OECloud platform (https://cloud.oebiotech.com/task/, accessed on 5 March 2024). The datasets have been deposited in the National Center for Biotechnology Information database (https://www.ncbi.nlm.nih.gov/sra, accessed on 5 March 2024) under the accession number PRJNA1228422.

### 3.19. Statistics

All data in this experiment were expressed as mean ± standard deviation (X ± S) and GraphPad Prism9 was used for significance analysis. A *t*-test was used to compare the two independent samples, one-way ANOVA was used to compare multiple samples and two-way ANOVA (or mixed model) was used to compare multiple groups and p≤0.05 was considered statistically significant. In all graphs, p≤0.05 was marked as *, p≤0.01 was marked as **, p≤0.001 was marked as ***, p≤0.0001 was marked as ****, and n≥3; ns, not significant.

## 4. Discussion

In recent years, the fertility rate has been declining, and multiple reproductive-related diseases have aroused widespread attention. Testicular torsion has become a concern for many people. If left unchecked, it can pose a significant threat to male fertility and have long-term consequences [34]. Our studies confirmed that testicular IRI triggered significant apoptosis and inflammation, as evidenced by elevated Cleaved Caspase-3, Cleaved PARP, Bax/Bcl-2 ratios, and NLRP3 levels. Additionally, testicular cell count and sperm viability decreased, accompanied by a decline in BTB-related factors (ZO-1, Occludin, and α-SMA), likely due to increased ROS [35]. To validate the results from our in vivo experiments, we conducted in vitro studies using a mouse Sertoli cell OGD/R model. The in vitro experiments revealed a decrease in cell viability and an increase in oxidative stress levels in OGD/R-treated cells.

*H19* is an imprinted gene and a long non-coding RNA. In the past, research on *H19* mainly focused on its role in tumor cell proliferation [36]. In recent years, its elevation following hypoxia has drawn attention due to its capacity in IRI. In microglial cells, *H19* forms a competing endogenous RNA network with miR-21 and PDCD4 to regulate the balance of NLRP3/6 inflammasomes29. In myocardial I/R, *H19* acts as a competing endogenous RNA for miR-877-3p, inhibiting Bcl-2 expression and participating in the regulation of mitochondrial apoptosis [37]. *H19* can also alleviate or exacerbate IRI in the heart and brain by regulating factors such as PPARα and PTEN [38,39]. We detected a significant increase in *H19* expression after testicular IRI. Using *H19* mice and si-*H19* TM4 cells, we demonstrated that *H19* suppression reduced apoptosis, inflammation (NLRP3, Bax/Bcl2), and ROS levels while restoring BTB proteins like ZO-1 in si-*H19*+OGD/R TM4 cells. Conversely, *H19* over-expression exacerbated cell damage and increased apoptosis following OGD/R.

Through transcriptome sequencing of three groups of TM4 cells (CTRL, OGD/R, si-*H19*+OGD/R), we found that the TGF-β signaling pathway, mineral absorption, alcoholic liver disease, Hippo signaling pathway, and other pathways highly enriched in KEGG all pointed to the AKT signaling pathway. Therefore, we examined the protein expression of AKT and phosphorylated AKT and preliminarily found that the elevation of *H19* after I/R was accompanied by the overactivation of the PI3K/AKT pathway. In mature Sertoli cells, PI3K/AKT can regulate protein synthesis and disrupt the BTB structure by affecting the Sertoli cell cytoskeleton [40]. It has also been reported in trophoblast cells that *H19* promotes trophoblast cell invasion and autophagy by activating the PI3K/AKT/mTOR pathway, thereby reducing cell viability [41].

To investigate the downstream factors of AKT and elucidate the mechanisms through which *H19* exerts its effects via AKT, we used the AKT activator SC79 in the si-*H19*+OGD/R group. Previous studies have shown that, in intestinal epithelial cells, *H19* disrupts and suppresses the transcription and translation of ZO-1 and E-Cadherin through miR-675, leading to dysfunction of the intestinal epithelial barrier [42]. Berberine (BBR) can restore intestinal epithelial barrier function by inhibiting lncRNA *H19* [43]. Therefore, we wondered whether BTB-related factors would change. We examined the expression of ZO-1, and the results show that the expression of ZO1 reverted to its normal level following the suppression of *H19*.

Additionally, in our quest to understand the deeper mechanism by which the PI3K/AKT pathway affects ZO-1, we retrieved mTOR, which are often influenced by AKT in various contexts. mTOR, the mammalian target of rapamycin, is divided into mTORC1 and mTORC2. mTORC1 promotes BTB remodeling and reorganization both in vivo and in vitro, while mTORC2 tightens the BTB. They are expressed at different stages of the spermatogenic cycle and control BTB dynamics [44]. mTORC is involved in the maintenance and reorganization of BTB and also plays a role in cellular energy metabolism, being a crucial regulator in spermatogenesis [45]. However, further investigation is needed to determine whether *H19* indeed affects mTOR and, consequently, ZO-1 expression through the PI3K/AKT pathway during testicular I/R. In addition, the relationship between *H19* and other factors, such as ROS and inflammatory cytokines, also warrants further investigation.

In conclusion, our study identifies *H19* as a critical mediator of testicular IRI. *H19* deficiency alleviated OGD/R-induced cell apoptosis and inflammation and restored ZO-1 expression, and the latter may be due to the stabilization of AKT phosphorylation, highlighting its potential as a therapeutic target. Targeting *H19* or the PI3K/AKT pathway may help mitigate IRI-induced testicular damage, although further preclinical and clinical validation is necessary to ensure safety and efficacy.

## Data Availability

Data are contained within the article and Supplementary Materials.

## Supplementary Materials

The following supporting information can be downloaded at: https://www.mdpi.com/article/10.3390/ijms26052134/s1.
