# Supplementary material for: Inhibition of LncRNA H19 Attenuates Testicular Torsion-Induced Apoptosis and Preserves Blood–Testis Barrier Integrity
Source: Int J Mol Sci. 2025 Feb 27;26(5):2134. doi: 10.3390/ijms26052134 (PMC11899958; doi:10.3390/ijms26052134)
Supplement: Supplementary file 1 [file ijms-26-02134-s001.zip › ijms-3465090-supplementary.pdf]

Supplementary Figure

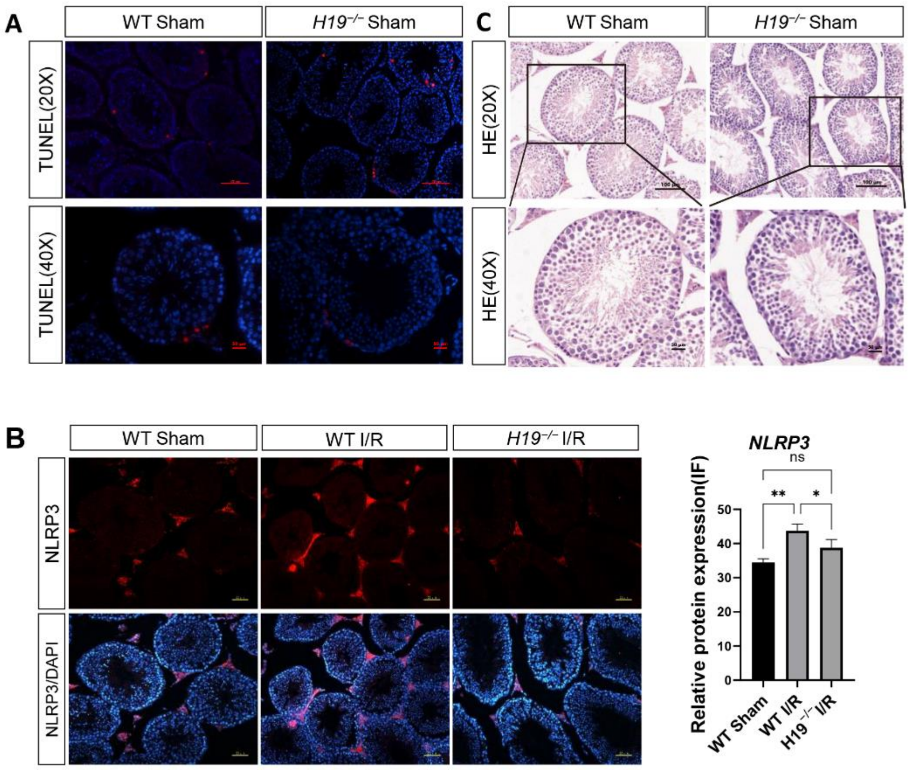

Figure S1. (A) TUNEL staining in the WT Sham group and the *H19*$^{-/-}$ Sham group under 20× and 40× magnifications. **(B)** The expression and quantification of NLRP3 in the WT Sham and *H19*$^{-/-}$ Sham mice were analyzed by immunofluorescence (NLRP3 is localized in the cytoplasm.). Scale bars=50 μm,100 μm. **(C)** HE staining in the WT Sham group and the *H19*$^{-/-}$ Sham group under 20× and 40× magnifications.

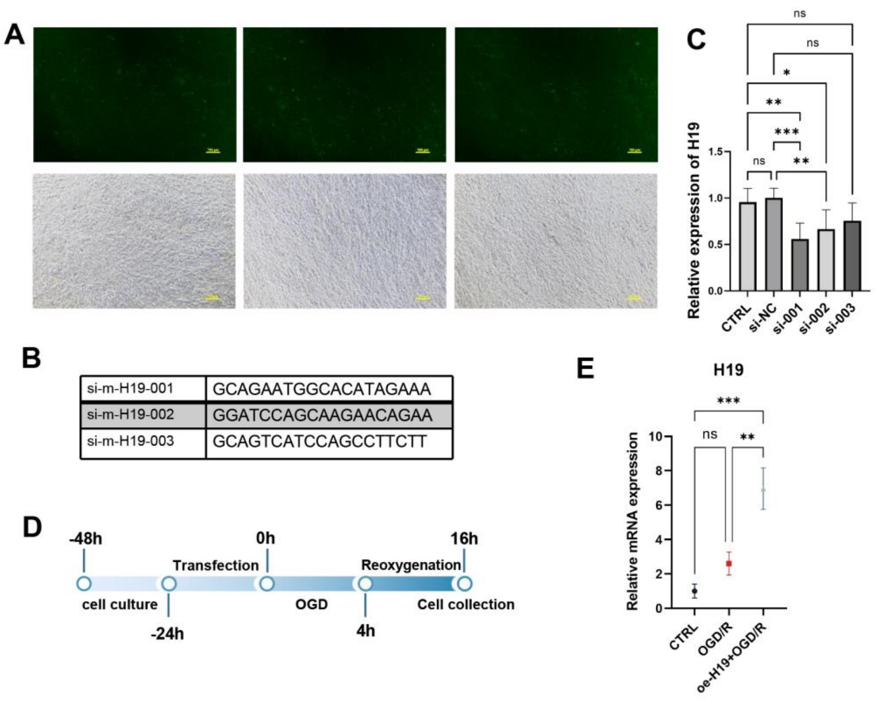

Figure S2. Transfection of si-*H19* and oe-*H19* and modeling of OGD/R. **(A)** Representative images of TM4 cells transfected with small interference FAM; n=3 per group, Scale bars=100μm. **(B)** Design sequence of *H19* siRNA. **(C)** qPCR analysis of three siRNAs transfected in TM4 cells. **(D)** Timeline of cell transfection and modeling. **(E)** In TM4 cells, the expression of H19 in the CTRL, OGD/R, and oe-H19+OGD/R groups.

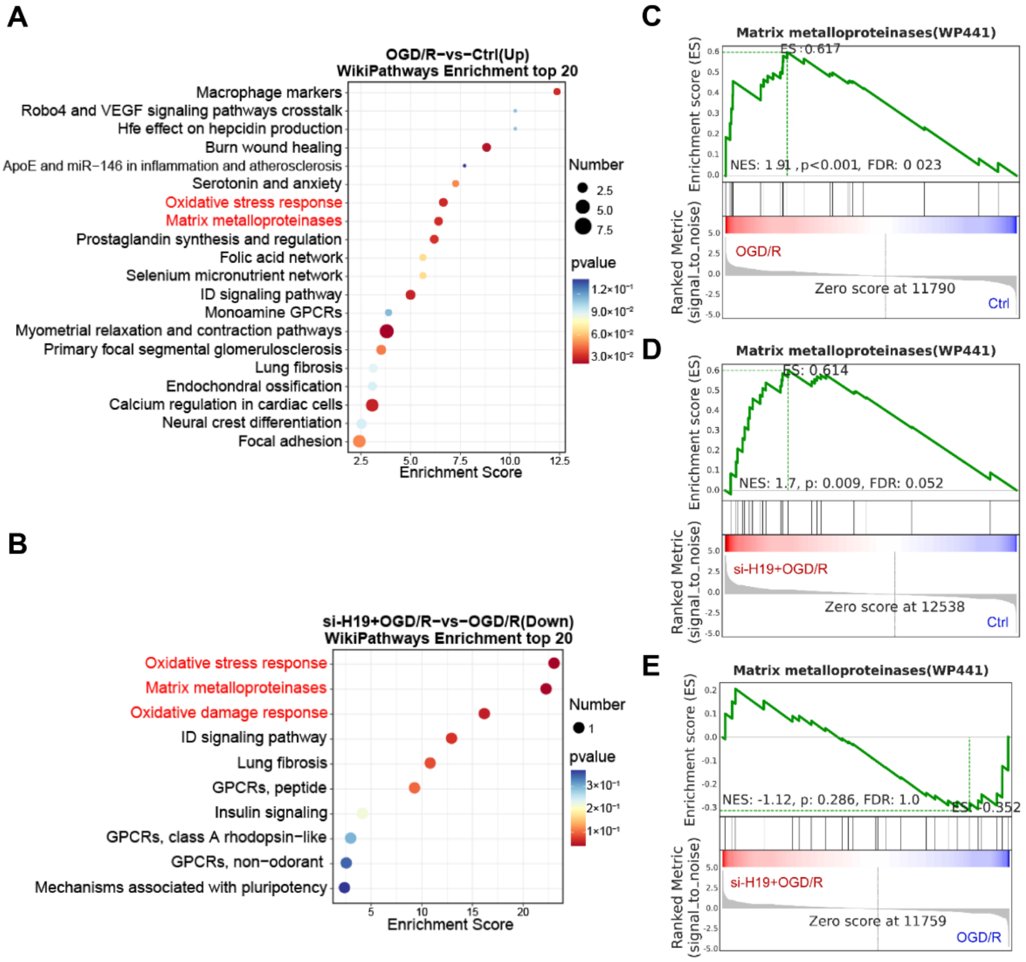

Figure S3. Oxidative stress and matrix metalloproteinase analysis. **(A,B)** Wiki pathways enrichment and comparison between OGD/R and CTRL groups, as well as si-*H19*+OGD/R and OGD/R. **(C-E)** GSEA analysis of matrix metalloproteinases in comparisons among three sample groups.

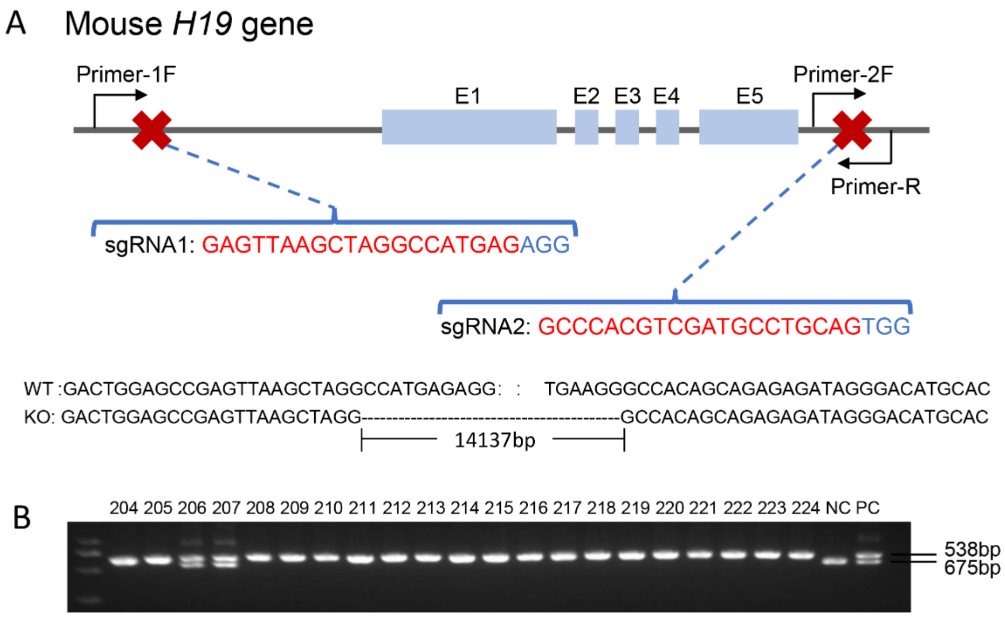

Figure S4. The construction of *H19* knockout mice. **(A)** Designing sgRNA and primers, as well as the genotyping sequences for WT (wild-type) and KO (knockout) mice. **(B)** *H19*$^{+/-}$ and *H19*$^{-/-}$ mice were genotyped by RT-qPCR. PC refers to the *H19*$^{+/-}$ genotype control; NC refers to the WT genotype control. Among these, samples 214, 215, 221, and 223 were used in our experiments.
